# Comparing risk environments for HIV among people who inject drugs from three cities in Northern Mexico

**DOI:** 10.1186/s12954-018-0225-y

**Published:** 2018-05-18

**Authors:** Angelica Ospina-Escobar, Carlos Magis-Rodríguez, Fatima Juárez, Dan Werb, Sergio Bautista Arredondo, Rubén Carreón, María Elena Ramos, Steffanie Strathdee

**Affiliations:** 1 El Colegio de Mexico, Mexico City, Mexico; 2 Centro Nacional para la Prevención y Control del SIDA (CENSIDA), Mexico City, Mexico; 3 Division of Global Public Health, University of California, San Diego, USA; 4 Instituto Nacional de Salud Pública, Cuernavaca, Mexico; 5 Programa Compañeros AC, Ciudad Juarez, Mexico

**Keywords:** PWID, HIV, Risk environment, Northern Mexico

## Abstract

**Background:**

A large body of research has investigated the rise of injection drug use and HIV transmission in Tijuana and Ciudad Juarez (CJ). However, little is known about the dynamics of injecting in Hermosillo. This study compares drug-related behaviors and risk environment for HIV of people who inject drugs (PWID) across Tijuana, CJ, and Hermosillo to identify factors that could explain differences in HIV prevalence.

**Methods:**

Data from Tijuana belong to a prospective study (El Cuete IV). Data from Hermosillo and Ciudad Juarez belong to a cross-sectional study. Both studies collected data in places where PWID spend time. All participants completed quantitative behavioral and serological testing for HIV. Datasets were merged using only comparable variables. Descriptive statistics tests were used to compare sociodemographic and behavioral characteristics of people who inject drugs PWID sampled in each city. A logistic regression model was built to identify factors independently associated with the likelihood of reporting receptive syringe sharing in the past 6 months.

**Results:**

A total of 1494 PWID provided data between March 2011 and May 2012. HIV prevalence differed significantly between participants in Tijuana (4.2%), CJ (7.7%), and Hermosillo (5.2%; *p* < 0.05). PWID from Hermosillo reported better living conditions, less frequency of drug injection, and lower prevalence of syringe sharing (*p* < 0.01). PWID from CJ reported a higher prevalence of syringe sharing and confiscation by police (*p* < 0.01). In a multivariable logistic regression model, living in Hermosillo compared to Tijuana (adjusted odds ratio [AOR] = 0.42, 95% confidence interval [CI] 0.29–0.61) and being female (AOR = 0.61, 95% CI 0.45–0.83) were protective against syringe sharing. Having used crystal meth (AOR = 1.62, 95% CI 1.24–2.13, *p* = 0.001), having experienced syringe confiscation by police in the last 6 months (AOR = 1.78, 95% CI 1.34–2.40), and lower perception of syringe availability (AOR = 2.15, 95% CI 1.59–2.91) were significantly associated with syringe sharing (*p* < 0.05).

**Conclusions:**

Differences in HIV prevalence across cities reflect mainly differences in risk environments experienced by PWID, shaped by police practices, access to injection equipment, and dynamics of drug markets. Findings highlight the importance of ensuring sterile syringe availability through harm reduction services and a human rights approach to drug harms in northern Mexico and to generate better understanding of local dynamics and contexts of drug use for designing proper harm reduction programs.

## Background

In the past two decades, Mexico has experienced an increasing prevalence of illegal drug use, most notably in the country’s northern border region. For example, according to the National Survey of Addictions, the proportion of people who reportedly used any illegal drug at least once in last year almost quadrupled, from 0.8% in 2002 [1] to 2.7% in 2016 [2]. The prevalence of lifetime illegal drug use in the northern region increased from 3.7% in 1988 [1] to 7.5% in 2012 [3]. By 2014, the state of Sonora experienced one of the highest incidence rates of drug use with 229.9 by 100,000 inhabitants [4].

As reported by the National Center for HIV/AIDS control and Prevention (CENSIDA), the northern cities of Ciudad Juarez (state of Chihuahua), Tijuana (state of Baja California), and Hermosillo (state of Sonora) are home to the largest population of people who inject drugs (PWID) in Mexico [5], representing an estimated 44% of the total Mexican population of PWID (*N* = 53,284).

Tijuana and Ciudad Juarez have a well-documented history of drug use and trafficking and its impact on public health [6–8]. CENSIDA (2010) estimated that roughly 12,000 PWID reside in Tijuana [5], among whom HIV prevalence is estimated at 4.2% [5]. In Ciudad Juarez, there are an estimated 10,000 PWID [5], among whom HIV prevalence is estimated at 7.7% [5].

By contrast, little is known about the dynamics of injection drug use in Hermosillo, Sonora, a city located 287 km from Tucson, AZ, where epidemiologic surveillance suggests an increasing prevalence of both HIV infection [8] and injection drug use. Mexico’s National Epidemiological Surveillance System for Addiction reports that, while in 2007, 15.0% of drug users in drop-in centers in the state of Sonora reported heroin as the primary drug of use [9], in 2009 that proportion had increased to 24.6% [10]. In 2012, in the state of Sonora, 41.2% of drug users attending addiction treatment reported crystal meth as the main drug of impact, with heroin reported by 19.6% of those in treatment [11].

Available data also show that in northern Mexico, the state of Sonora reported the highest rise in the volume of people with patterns of abusive drug use. In 2015, Sonora counted for the 48.3% of the population in rehabilitation centers of northern Mexico [12]. CENSIDA reported that the rate of incidence of injected drug use by 100,000 inhabitants in Sonora rose from 6.3 in 2006 to 53.8 in 2014, while in Baja California in this same period, dropped from 133.8 to 19.7 and in Chihuahua from 71.3 to 2.6 [4, 13]

Additionally, Sonora is also one of the states in Mexico with the most rapid escalation of HIV incidence related to injection drug use: data from the Secretary of Health in Mexico indicate that before 2000, this mode of transmission represented 4.8% of all new HIV cases in the city of Hermosillo [14], while between 2000 and 2014, injection drug use was responsible for 23.1% of new HIV cases, an increase of over 340% [15].

The rapid expansion of HIV and injection drug use in Hermosillo could be associated with a variety of factors. First, it may be associated with recent changes in local drug markets and transnational drug trafficking routes [16]. Trends of drug use in Sonora demonstrate that heroin use rapidly increased since 2000 and crystal methamphetamine use since 2005, which coincides with the period when Mexico started to become a lead exporter of both heroin and crystal meth [16]. Second, official data show a dramatic rise of violence in the state, for example, the rate of homicides rose 74% between 2010 and 2015 [17]. Third, there has been a reduction in support for a range of HIV prevention interventions in Hermosillo, including needle exchange programs, free access to methadone maintenance therapy and HIV testing at PWID meeting places. This reduction is mainly related to the end of funding from the Global Fund to Fight HIV, Tuberculosis, and Malaria, which previously funded HIV prevention activities in Hermosillo [18].

Given this emerging public health crisis, understanding the characteristics of PWID and drug-related behaviors in Hermosillo, and how they resemble more established epidemics of injecting and HIV in other settings in Mexico’s northern border region (i.e., Tijuana and Ciudad Juarez), could inform evidence-based HIV prevention strategies in Mexico. We, therefore, sought to compare drug-related behaviors of PWID from Tijuana, Ciudad Juarez, and Hermosillo and to identify individual and social factors that could explain differences in HIV prevalence among PWID.

## Methods

### Sampling and recruitment

We employed multiple sources of quantitative data from two different projects. Data from Tijuana are part of a prospective study conducted by the University of California at San Diego (UCSD) and in operation since 2005 (i.e., Proyecto El Cuete IV) [6]. Participants were recruited through targeted sampling, consisting of street-based outreach in ten different neighborhoods where PWID were known to spend time and where they were invited to participate by outreach workers [19, 20]. Data from this cohort were obtained from March 2011 to May 2012.

Data from Hermosillo and Ciudad Juarez were collected as part of a cross-sectional survey supported by the Global Fund to Fight AIDS, Tuberculosis and Malaria (Round 9) to evaluate HIV risk among PWID in Mexico’s northern region. The questionnaire was based on the Proyecto El Cuete questionnaire. In Hermosillo and Ciudad Juarez, the survey was conducted between January and June 2012 in places that PWID were known to frequent (hotspots), following time-location-sampling methodology. This involved a previous process of mapping the sites and enumerating the population in each one of them to select the meeting points with greater attendance of PWID [21]. In these cities, the sampling frame was elaborated from a list of places built by local organizations that implement harm reduction activities. Each spot was visited by the field team and PWID attendees were counted. Spots were classified into small ones (with an attendance of fewer than 100 people) and large ones (with an attendance of more than 100 people), and a sample size proportional to each type of spot was assigned. The selection of small sites was done through systematic sampling and all large sites were included. Within each site, potential participants were randomly selected [21].

**Fig. 1:**
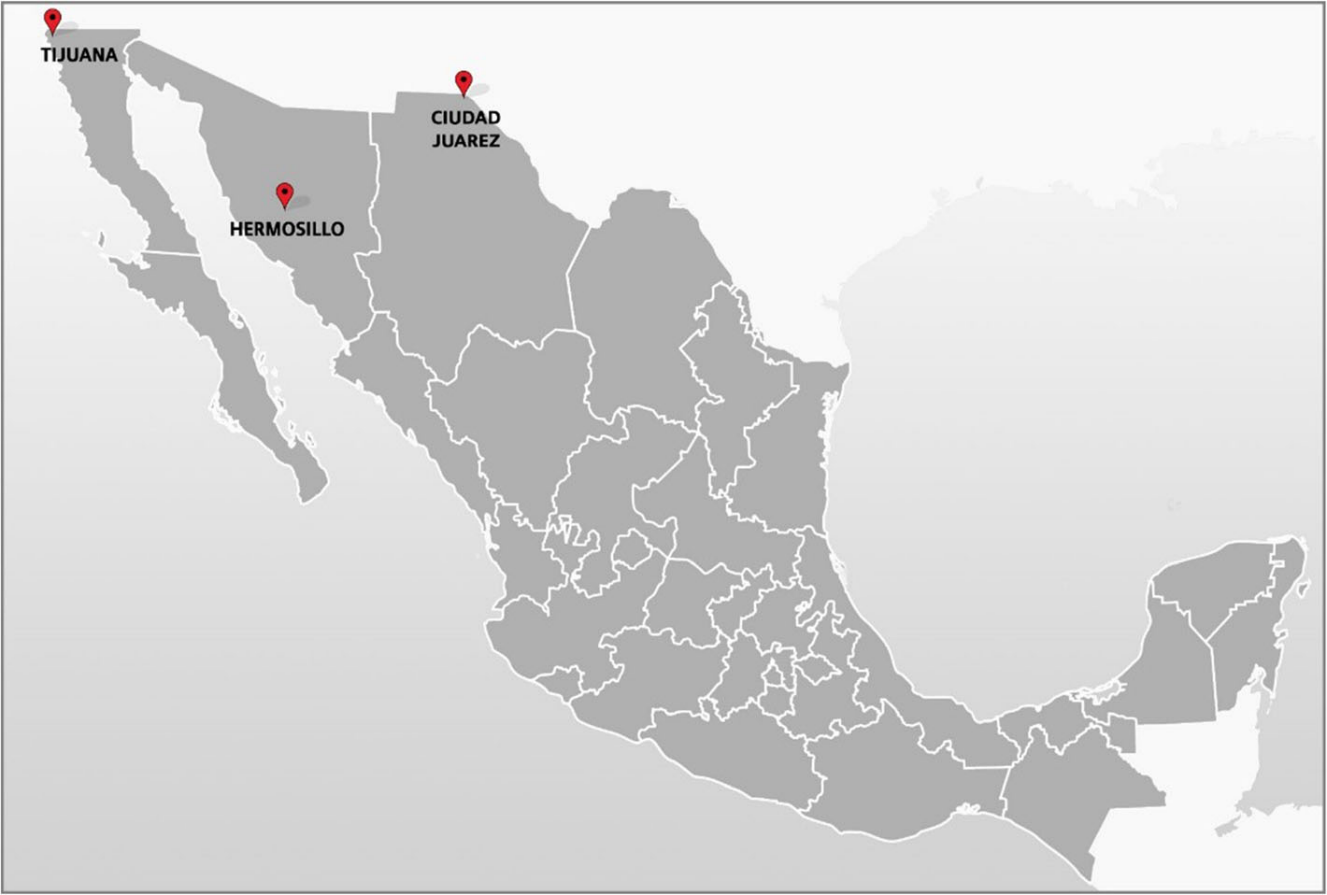
Location of Tijuana, Hermosillo and Ciudad Juarez in Mexico

In the three cities (Fig. 1), potential respondents were screened to verify if they met the eligibility criteria. Screening began with the provision of general information about the study aims and procedures. Potential participants were asked for verbal consent before beginning the screening interview. Ineligible individuals were offered free condoms, information, and referrals for HIV testing. All participants signed an informed consent form. Study participants were at least 18 years old, reported having injected drugs at least once during the last 3 months and reported being permanent residents of the selected cities. Participants from Tijuana received US$20. Consistent with local research protocols, participants from Hermosillo and Ciudad Juarez received prevention kits with injecting paraphernalia. The Institutional Review Boards of the UCSD School of Medicine and the Mexico National Institute of Public Health (INSP) approved the study [6, 21].

Data were collected by trained interviewers who also had experience working with PWID. The survey includes a range of questions related to sociodemographics and sexual and drug use risk behaviors, history of drug use and treatment, exposure to health facilities, and other aspects of the physical and social risk environments that may increase the probability of HIV acquisition among participants. In average, it took 20 min to complete the survey. While in Tijuana, the questionnaire was administered in English or Spanish by bilingual interviewers, in Ciudad Juarez and Hermosillo, it was conducted only in Spanish.

In the three cities, serologic testing for HIV infection was conducted. In Tijuana, participants received pre- and post-test counseling. In Hermosillo and Ciudad Juarez, participants did not receive results. Serologic testing involved blood specimens collected via fingerstick and venipuncture in line with standard clinical practice by trained phlebotomists who were experienced in obtaining blood from PWID with scarred veins. Reactive rapid tests were repeated. Participants receiving a second reactive rapid test were considered positive and referred to nearby municipal health clinics for free care under Mexico’s universal health system (e.g., CAPASITS).

Datasets of each city were merged using only comparable variables. Certain nominal variables were recodified to improve comparability.

### Conceptual framework

This analysis is guided by the risk environment framework [18], which posits the need to shift from individualistic approaches of risk behavior towards an understanding of how structural and environmental conditions shape an individual’s vulnerability to HIV acquisition. Risk environment is defined as the space—whether social or physical—in which four types of environmental influence factors—physical, social, economic, and policy—interact to two micro and macro characteristics, increasing the chances of drug-related harm [22, 23]. While the micro-risk environment focuses on personal decisions as well as the influence of community level norms and practices. The macro-risk environment seeks to capture structural factors, such as laws, military actions, economic conditions, and institutional settings.

This framework highlights the multilevel and contextualized nature of HIV risk and focuses on interactions between risk factors exogenous to the individual, rather than endogenous factors, such as risk practices, age, or sex [22].

### Variables of interest

We included variables that correspond to the different domains outlined by the risk environment framework. We included city of residence as a measure of the meso-level policy, social, economic, and geographic environment that participants experience. For the social aspect of the risk environment, we included sex (male vs. female); age; migration status (born in the current city of residence vs. born anywhere else); level of educational attainment (completed secondary or more vs. partial secondary or less, which is the level to which education is compulsory in Mexico); and reporting at least one commercial sex partner during the last six months (yes vs. no). Micro-level variables assessing the economic environment were monthly income (less than US$212 vs. more). Drug-related variables were classified as assessing both social and economic variation given that they imply differences in individual drug use patterns and likely also reflect meso-level differences in local drug markets. Specific drug-related variables included: current frequency of drug injection (daily vs. less than daily) and use of crystal meth during the last 12 months (yes vs. no). Policy/political environment was assessed by variables related to interactions with criminal justice or public health institutions. These included having experienced syringe confiscation by police during the last 6 months (yes vs. no) and perceived syringe/needle availability (based on responses to the question “In the last 6 months, how easy or hard was it for you to get new, unused syringes when you injected drugs”; responses were dichotomized as “hard/very hard” vs. “easy/very easy”).

Finally, for the outcome variable participants were considered to have participated in receptive needle sharing if they responded “sometimes”, “often” or “always” to the question: “How often did you use a syringe that you knew or suspected had been used before by someone else?”.

### Statistical analyses

For descriptive analyses, we employed Cramer Alpha and Phi statistics tests to compare sociodemographic, behavioral, and context-related characteristics of the sampled individuals by city, due to their sensitivity in analyzing small size samples [24]. We then built an explanatory logistic regression model to identify factors independently associated with the odds of having receptively shared syringes in the past 6 months. The model was developed using an a priori design whereby variables with a significance level of *p* < 0.1% in univariate analysis were considered for inclusion in a multivariable model using the forward method. We analyzed multicollinearity between the predictor variables using the Pearson correlation test and we only included variables with a correlation lower than 0.3. We used the Hosmer and Lemeshow test to analyze the model’s goodness of fit. The statistical analysis was run using SPSS version 19 (SPSS, Chicago, IL).

## Results

### Sample characteristics

A total of 1494 people who injected drugs provided data for this analysis. Table 1 presents descriptive data. 44.8% (*n* = 670) of participants lived in Tijuana, 26.2% (*n* = 392) in Hermosillo, and 28.9% (*n* = 432) in Ciudad Juarez.

**Table 1:** Social risk environment variables of PWID by city. México, 2012

Social risk environment variables	Sample distribution by city			All (*N* = 1494)
	Tijuana (*n* = 670)	Cd. Juarez (*n* = 432)	Hermosillo (*n* = 392)	
Age (mean)***	38.9	38.8	29.2	35.7
Sex (%)***				
Women	34.8	25.9	7.9	25.2
Men	65.2	74.1	92.1	74.8
Civil status (%)***				
Single	42.5	43.8	65.3	51.1
Ever married	57.5	56.3	34.7	48.9
City of birth (%)***				
Born in the current city of residence	35.7	69.9	79.3	56.9
Born in a different city	64.3	30.1	20.7	43.1
Educational attainment (%)***				
Uncompleted secondary or less	60.1	55.9	13.0	47.6
Complete secondary or above	39.9	41.4	86.0	52.4
Have had at least once commercial sex partner during the last 6 months (%) (***)				
Yes	28.2	26.2	14.3	24.0%
No	71.8	73.8	85.7	76.0%
Monthly income (%) (Mex. pesos)***				
Less than $3499	74.3	70.1	39.9	64.6
More than $3499	25.7	29.9	60.1	35.4
Frequency of receptive sharing needles (%) ***				
Never	29.0	28.9	41.6	32.3
Less than half of the times	37.9	33.3	29.6	34.4
Half of the times	10.6	14.4	5.6	10.4
More than half of the times	14.9	15.5	16.3	15.5
Always	7.6	7.9	6.9	7.5
Distribution	100%	100%	100%	100%

Note: Significant differences by city of residence ***p < 0.01;

Regarding the outcome variables, HIV prevalence found in the sample was 4.2% in Tijuana, 5.2% in Hermosillo, and 7.7% in Ciudad Juarez. Participants from Hermosillo reported a lower prevalence of frequency of needle and syringe sharing during the prior 6 months (31.0%; *n* = 229), compared to those from Ciudad Juarez (71.1%; *n* = 307; *p* < 0.05) or Tijuana (71.0%; *n* = 476; *p* < 0.05).

Variables related to the social risk environment indicated the median age of participants was 35.4 (standard deviation [SD] 10.0). Participants from Hermosillo were significantly younger than participants from the other two cities (29.2 vs. 38 years old; *p* < 0.01). Most participants were born in the current city of residence (56.9%; *n* = 847), but a significantly larger proportion of participants in Tijuana reported having been born in a different city (64.3% [*n* = 431] vs. 30.1% [*n* = 130] in Ciudad Juarez; *p* < 0.01). By contrast, a significantly smaller proportion of those living in Hermosillo reported having been born in a different city (20.7% [*n* = 80]; *p* < 0.01).

### Sexual practices

In relation to sexual practices, 80.6% of participants reported having sex during the 6 months prior to the survey; among them, almost one out of four participants reported having had a commercial sex partner. Participants from Hermosillo reported less prevalence of commercial sex (14.3%) compared to those from Tijuana (28.2%) and those from Ciudad Juarez (26.2%).

Tijuana was the city with the lowest frequency of condom use with casual and commercial partners (35.7 and 43.6%, respectively; *p* < 0.01). Participants from Hermosillo reported a significantly lower frequency of condom use compared to those from Ciudad Juarez (48.2% of participants in Hermosillo reported having used condoms at least half of times that had sex with casual and commercial partners while in Ciudad Juarez 60.1% reported having used with casual partners and 68.4% with commercial partners).

To better understand the sexual practices and its relation to the differences found in HIV prevalence among cities, we compared the proportion of men who inject drugs who reported having sex with men and its differentials by city. The prevalence of men who reported having had sex with other men was 6.0% (*n* = 67/1118). Tijuana was the city with the highest proportion of MSM-PWID (7.1%), while in Ciudad Juarez and Hermosillo, the proportion was 5.3%.

### Drug-related dynamics

On the other hand, with respect to drug use patterns across the three cities, which are likely indicative of differing social and economic risk environments, a significantly larger proportion of participants from Hermosillo reported cocaine as their drug of initiation compared to participants in other cities (8.4%; *n* = 33 vs. 6.9%; *n* = 30 in Juarez and 5.2%; *n* = 35 in Tijuana; *p* < 0.01). A significantly (*p* < 0.01) larger proportion of participants from Tijuana and Ciudad Juarez reported having initiated drug use with inhalants (8.8%; *n* = 59 and 14.4%; *n* = 62, respectively) and heroin (9.4%; *n* = 63 and 12.0%; *n* = 52, respectively) in comparison with those from Hermosillo (7.4%; *n* = 29 of whom reported having initiated drug use with inhalants and 3.6%; *n* = 14 with heroin).

Mean age for onset of illegal drug use was 14.9 years with significant differences between cities (*p* < 0.05). Those from Hermosillo reported an earlier onset of illegal drug use (14.3 years), while those from Ciudad Juarez reported a later onset (15.5 years).

Regarding current dynamics of drug use, most participants reported using more than one type of drug at a time (61.8%). Nevertheless, the main drug currently injected by participants was heroin (77.2%; *n* = 1143), in a frequency of daily or more (87.7%; *n* = 1310). Also, most participants reported using other drugs in combination with heroin (61.8%; *n* = 923). Cocaine in isolation was reportedly used by 14% of participants from Tijuana (*n* = 94), 37.7% (*n* = 163) of those from Ciudad Juarez, and by 42.1% (*n* = 165) of those from Hermosillo (*p* < 0.00). By contrast, crystal methamphetamine use was reported by a minority of participants from Ciudad Juarez (7.2%; *n* = 31) and by a slight majority of participants from Tijuana (53.0%; *n* = 355; *p* < 0.05). Furthermore, while almost all participants from Tijuana and Ciudad Juarez reported injecting drugs once a day or more (96.4%; *n* = 646 and 91.2%; *n* = 394, respectively), only two thirds of participants from Hermosillo reported injecting at least daily (68.9%; *n* = 270) (*p* < 0.05).

### Policy context

With respect to the political/policy risk environment participants experienced, Table 2 presents data on participant interactions with criminal justice and public health institutions. As can be seen, one third of participants reported experiencing syringe/needle confiscation by police during the prior 6 months, though proportions differed significantly across cities. Specifically, 9.3% (*n* = 62) of participants in Tijuana reported syringe or needle confiscation by police, compared with approximately 40% of participants in both Ciudad Juarez (40.3%; *n* = 174) and Hermosillo (39.0%; *n* = 153; *p* < 0.01).

**Table 2:** Socioeconomic and political risk environment experienced by PWID drugs by city. Mexico, 2012

Socio-economic and political risk environment variables	Sample distribution by city			All (*N* = 1494)
	Tijuana (*n* = 670)	Cd. Juarez (*n* = 432)	Hermosillo (*n* = 392)	
Socioeconomic risk environment variables				
Dynamics of illegal drug use during the last 12 months				
Drugs used (%)**				
Heroin	63.4	97.9	77.6	77.2
Cocaine	14.0	37.7	42.1	28.2
Heroin and cocaine together	14.6	28.7	17.6	19.5
Crystal meth	53.0	7.2	53.3	39.8
Crystal meth and heroin together	56.3	2.3	16.1	30.1
Other drugs	0.0	0.5	2.1	0.7
Type of user according with the number of drugs used (%)***				
Had used only one type of drug	30.9	49.4	37.1	38.2
Had used more than one type of drug (polysubstance user) (%)***	69.1	50.6	62.9	61.8
Frequency of drug injection (%)**				
Less than once a day	3.6	8.8	31.1	12.3
Once a day or more	96.4	91.2	68.9	87.7
Political/policy risk environment variables				
Police harassment (%)***				
Police have confiscated needles at least once during the last 6 months	9.3	40.4	39.7	26.2
Perception of syringe availability (%)***				
Very easy	35.9	13.2	9.7	22.5
Easy	44.7	52.5	73.7	54.6
Hard	18.2	29.9	14.3	20.6
Very Hard	1.2	4.4	2.0	2.3

Note: Significant differences by city of residence ***p < 0.01; **p < 0.05;

Participants from Ciudad Juarez reported the lowest perception of syringes availability, as 34.3% declared it is hard or very hard to get new syringes, while among Hermosillo participants, the proportion was 16.6%. Among participants from Tijuana, the proportion was 19.4% (*p* < 0.01).

### Factors associated with receptive syringe sharing

As shown in Table 3, in multivariable logistic regression analysis, receptive needle sharing was significantly associated with the frequency of drug injection (adjusted odds ratio [AOR] = 2.43, 95% confidence interval [CI] 1.52–3.90, *p* < 0.01), having monthly incomes higher than US$212 (AOR = 1.45, 95% CI 1.11–1.88, *p* = 0.006), having at least one commercial sex partner during the last 6 months (AOR = 2.22, 95% CI 1.60–3.09, *p* < 0.01), having used crystal meth during the last 12 months (AOR = 1.60, 95% CI 1.22–2.21, *p* = 0.001), having experienced syringe confiscation by police during prior 6 months (AOR = 2.15, 95% CI 1.59–2.91, *p* < 0.01), and perceiving syringe availability as hard or very hard (AOR = 1.82, 95% CI 1.36–2.44, *p* < 0.01). Factors that were negatively associated with needle sharing were living in Hermosillo compare to living in Tijuana (AOR = 0.40, 95% CI = 0.28–0.57, *p* < 0.01) and being female (AOR = 0.60, 95% CI = 0.44–0.82, *p* = 0.001).

**Table 3:** Multivariable analysis of factors associated with receptive needle sharing among PWID in Tijuana, Ciudad Juarez, and Hermosillo. México, 2012 (*N* = 1463)

Characteristic	Adjusted odds ratio	95% confidence interval	*p* value
Age	0.40	0.98–1.01	0.42 (ns.)
Sex (men vs. women)	0.61	0.45–0.83	0.002
Migration status (migrant vs. non-migrant)	1.04	0.80–1.34	0.77 (ns.)
Monthly income	1.45	1.11–1.87	0.006
City			
Tijuana (ref)	1	1	
Cd. Juarez	1.00	0.70–1.41	0.99 (ns.)
Hermosillo	0.42	0.29–0.61	< 0.01
Frequency of injection drug use	1.91	1.35–2.72	< 0.01
Have had crystal meth during prior 12 months	1.62	1.24–2.13	< 0.01
Have had at least once commercial sex partner during prior 6 months	2.22	1.61–3.11	< 0.01
Have experienced syringe confiscation by police during prior 6 months	1.78	1.34–2.40	< 0.01
Perception of syringe availability as hard or very hard	2.15	1.59–2.91	< 0.01

## Discussion

The noted differences in HIV prevalence among PWID in Tijuana, Hermosillo, and Ciudad Juarez could be interpreted as the result of the net-effects of individual characteristics, political context, and dynamics of local drug markets.

As such, the lowest HIV prevalence found in Tijuana could be associated with lower police harassment against PWID and the higher perception of syringes availability. In this context, despite the more individual vulnerability in terms of less educational level, less level of incomes, and more proportion of migration, it seems that the institutional setting operates as a cushion that protects individuals from risk behaviors, such as sharing syringes or having unprotected sex. It is also possible that the interaction with people from the USA provides more risk perception about HIV and less willingness for sharing injection equipment.

By contrast, the highest HIV prevalence found in Ciudad Juarez could be interpreted as a collateral effect of the war on drugs that creates a context of greater stigmatization and criminalization of drug users and greater control from criminal organizations. In this context, drug users are forced to rushed injections and altering their drug use dynamics to avoid police and gangs. Since syringes are perceived as non-available, PWID could be more willing to share their equipment as a means to get a “quick fix” avoiding police mistreatment and detention.

Hermosillo appears as an in-between scenario. The higher HIV prevalence found in Hermosillo compared to Tijuana may be related to the greater availability of white heroin and crystal meth binges. White heroin and its characteristics of injection has been associated with higher levels of HIV prevalence in the USA [25–27]. On the other hand, it has been shown how cocaine and crystal meth binges are correlated with higher prevalence of HIV among PWID [28, 29] as a result of several factors, among others, the more compulsive injecting behavior during a shorter time compared with heroin users’ injecting behavior (which is more permanent but less compulsive) and the practical considerations of planning, obtaining, and transporting sufficient sterile syringes to carry one through binge [26, 28]. Cocaine and crystal meth binges are also associated with unprotected sex because of the reported increase in energy, sexual arousal and performance, and atypical sexual behaviors linked to methamphetamine use [29]. In these data, participants from Hermosillo reported being more sexually active and have lower condom use compared to those from Tijuana and Ciudad Juarez.

Among participants in Hermosillo, a higher level of education and income, as well as better living conditions, appear to be protective against police harassment and suggest greater means to buy syringes. Nevertheless, local drug use dynamics (e.g., cocaine and crystal methamphetamine bingeing) likely generate a higher frequency of injecting and, by extension, risk for syringe sharing.

## Conclusion

This is the first study comparing data across three different cities in Mexico’s northern region. We have described micro and meso-level characteristics within each city that shape differentiated risk environments for HIV.

Tijuana shows a prevalence of HIV among PWID of 4.2%. Participants from this city reported higher levels of migration status and poorer socioeconomic conditions (i.e., lower educational attendance, lower incomes, higher involvement in commercial sex, and among men, higher prevalence of sex with other men). Most of participants from this city reported injecting more than once a day every day, and a majority (71.5%) reported using crystal meth and black tar heroin, with a scarce presence of white heroin. Despite their harsh living conditions, these participants reported the lowest prevalence of syringe sharing (33.1%), the lowest proportion of syringe confiscation by police (9.3%), and higher perception of syringe availability (80.6%).

By contrast, Ciudad Juarez displays the highest HIV prevalence (7.7%). Juarez, along with Tijuana, has a long history of drug smuggling and its prevalence of injecting drug use and heroin use is higher than the national level since the first epidemiological data, back to the 1990s. Juarez and Tijuana participants have a similar sociodemographic profile characterized by low monthly income, low educational attainment, and high involvement in commercial sex. In terms of drug use profile, Juarez participants are mainly black tar heroin users, with a high frequency of injection (mainly more than once a day). They declared the lowest prevalence of poly drug use in comparison with Tijuana and Hermosillo and a limited use of crystal meth (9.5%). These participants also declared the highest prevalence of syringe sharing (37.8%), the highest proportion of syringe confiscation by police (40.4%), and the lowest perception of syringe availability (65.7%).

In Hermosillo, data reveals an HIV prevalence among PWID of 5.2%. Data describes a different participants profile compared to those from Ciudad Juarez and Tijuana. PWID from Hermosillo have more level of schooling, declared in higher proportion having formal jobs, better income level, lower participation in commercial sex, and among men, less involvement in sex practices with other men. They also reported different drug use profile. Approximately 1 out of 20 declared using white heroin and almost half reported using brown heroin; they reported less intensive patterns of drug injection, as 31.1% declared injecting less than once a day, more diversity of drugs used at the same time, and lower frequency of syringe sharing.

Logistic regression model suggests that individual characteristics are important factors associated with syringe sharing, especially having had a commercial sex partner and the frequency of drug injection. The overlapping of sexual and injection risk behaviors suggests multiple pathways for HIV acquisition among the study population.

Firstly, findings demonstrated that syringe confiscation increases 64% the likelihood of receptive needle sharing despite adjusting for variables such as the city of residence and individual characteristics. 26.2% of participants reported syringe confiscation, but it is not distributed uniformly across the population. The higher proportion of participants reporting syringe confiscation in Ciudad Juarez is likely related to the high levels of drug-related violence and the subsequent humanitarian crisis that the city has experienced since the Mexican “War on Drugs” was launched in 2006 [16]. Specifically, the presence of military forces likely exposes PWID to higher levels of harassment and the possibility of human rights violations, including the right to free mobilization and access to sterile syringes. Previous studies had shown the importance of police practices on HIV prevalence and other poor health outcomes among PWID [6, 7, 30, 31].

Secondly, syringe availability appears as a major driver of syringe sharing. Those with the poorer perception of syringe availability had 2.22 times more likelihood of syringe sharing than those with more positive perception. Ciudad Juarez was the city with the poorer syringe availability perception, which could be related to the higher rate of confiscation by police.

Thirdly, the differences found in drug use profile among the three cities suggest that among PWID, the choice of drug and the patterns of drug use could not be as variable as it has been reported [30], and it could be associated with the control enforced by criminal organizations in local drug markets. For example, the lower crystal meth use found in Ciudad Juarez suggest a local drug market mostly restricted to black tar heroin and cocaine. Tijuana and Hermosillo appear to have a more diverse drug market, with greater availability of cocaine, white heroin, and crystal meth. These differences could be attributed to the different criminal organizations that control Hermosillo and Tijuana (Cartel de Sinaloa) and Ciudad Juarez (Cartel del Golfo) [16]. Cartel de Sinaloa is the main distributor of crystal meth and main producer of Mexican white Heroin, while Cartel de Juarez controls the smuggling of black tar heroin and cocaine along the border with the USA [16]. This has important implications for the risk of HIV and Hepatitis C virus infections and the delivery of harm-reducing interventions (i.e., despite the increase rate in methamphetamine use in Mexico, treatment options for dependency remain woefully inadequate).

### Public policy implications

Our data suggest that, increasing access to sterile syringes is critical to addressing HIV risk among PWID in Mexico’s northern region. It is needed to renew investment in public health interventions to respond effectively to drug-related harms. Specifically, there is an urgent need to enhance the institutional response to HIV among PWID in Mexico by promoting community-based harm reduction programs and peer-leading interventions and by creating linkages between the PWID population, community-based organizations, and governmental institutions to ensure that the human rights of PWID are promoted and protected.

While Mexico has already initiated a process of legislative reforms to move towards a more comprehensive drug policy, the implementation of these changes has been slow. As these reforms are meaningfully adopted by governments at all levels, efforts must be made to ensure their accordance with a human-rights-based approach that seeks to strengthen the ability of civil society to respond to drug-related harms in an effective and respectful manner.

Success in reducing the incidence of HIV infection among injecting drug users will only be realized by providing relevant interventions in a timely fashion, considering the specific dynamics of drug use and policing practices. The outbreak of HIV infection among PWID in Hermosillo during the last decade cannot be attributed to any single environmental or individual factor; as such, it is important to enhance the understanding of how different level of characteristics interacts to build risk environments for HIV in each local scenario. The mix between quantitative and ethnographic data could help to improve the understanding of who, how, why, and where of the HIV infection [32].

### Study limitations

It is difficult to know to what extent the samples are representative of the broader population of PWID in the selected cities. As data were collected where PWID gathered, it is possible that the samples had higher levels of homogeneity among participants compared with the overall populations of PWID in each city. As such, findings cannot be generalized to the broader population of PWID in Mexico. The cross-sectional nature of the survey data also limits our capacity to detect causal associations. As such, this study is restricted to highlighting the associative relationships between variables. More research is needed to identify the causal pathways and the complex set of relationships that influence syringe sharing and HIV prevalence among sampled PWID. Additionally, data were collected at different times at each city (from March 2011 to May 2012 in Tijuana, January to March 2012 in Ciudad Juarez, and from May to June 2012 in Hermosillo). This would imply a potential bias in the trends of drug use and exposure to different interventions assuming substantial changes in drug use patterns, drug availability, or intervention implementation between the two data collection periods (i.e., 2011 vs. 2012). However, political and policing contexts did not change greatly between the interval when data were collected in the three sites, nor have there been reports of substantial changes in drug-related trends across the data collection periods.

Data from Ciudad Juarez and Hermosillo were collected using time-location sampling and the same standards were applied in data collection methodology. Data from Tijuana are from a community-recruited prospective study, and these differences in data collection may affect their comparability.

Even though data were collected from 2011 to 2012, this is the latest available information about PWID in Hermosillo and Ciudad Juarez, suggesting the findings are still relevant for the regions and populations under study. In Tijuana, UCSD collects data continuously among PWID as a part of El Cuete Project. A recent study conducted among PWID in Tijuana found similar sociodemographic characteristics, drug use dynamics, and HIV prevalence than those reported in this study [33]. In order to generate more accurate and pertinent data surrounding PWIDs in Mexico, consistent epidemiological surveillance is needed in cities with disproportionately larger PWID populations.

Given the scarcity of data available, there is an urgent need to better understand the mechanisms of HIV spread among PWID in Mexico’s northern region. These findings provide an important preliminary insight into the heterogeneity of PWID populations across northern Mexican settings, how risk environments shape HIV transmission among PWID in these settings, and how vulnerable drug-using populations in Mexico may be responding to pressures from drug-related violence. Our study’s findings also highlight important implications for the prevention of an emerging HIV epidemic in Hermosillo.

Local data and related conclusions are focused on the specific Northern Mexican cities under study. However, given the high level of cross-border mobility, specifically among people who use drugs, the lack of harm reduction programs in study sites suggest that HIV acquisition among PWID and their sexual partners is likely occurring at elevated rates on both sides of the border.
